# Informal carers’ experience and outcomes of assistive technology use in dementia care in the community: a systematic review protocol

**DOI:** 10.1186/s13643-019-1081-x

**Published:** 2019-07-03

**Authors:** Vimal Sriram, Crispin Jenkinson, Michele Peters

**Affiliations:** Health Services Research Unit, Nuffield Department of Population Health, University of Oxford, Richard Doll Building, Old Road Campus, Oxford, OX3 7LF UK

**Keywords:** Dementia, Informal carer, Assistive technology, Quality of life, Stress, Burden

## Abstract

**Background:**

Dementia is one of the greatest health and care priorities globally. Caring for persons with dementia is a challenge and often leads to negative psychological, physiological and financial consequences for informal carers (family members or friends). Many informal carers experience moderate to severe levels of burden. Advances in technology have the potential to assist persons with dementia and their carers, through assistive technology (AT) devices such as electronic medication dispensers, robotic devices and motion detectors. However, little is known about informal carers’ experience and the impact of these technologies on them. This review aims to investigate the outcomes and experience of carers of persons with dementia, who live at home and use AT.

**Method:**

MEDLINE, Embase, CINAHL, AMED, ALOIS, PsycINFO, Trial registries and OpenGrey databases will be searched for studies of any design that have investigated carer experience and/or outcomes of AT use for persons with dementia living at home. Manual searches from reference lists of relevant papers will also be undertaken. Outcomes of interest are carers’ self-reported outcomes (which include perceived burden, quality of life and wellbeing) and carer experiences (such as usefulness, benefits and disadvantages of AT and impact on caregiver/care receiver relationship). Two independent reviewers will screen identified papers with pre-defined eligibility criteria and extract data using a bespoke extraction form. Discrepancies will be resolved in discussion with a third reviewer. A synthesis of eligible studies and summary will be provided.

**Discussion:**

A systematic review of quantitative, qualitative and mixed methods evidence of informal carers’ experience of AT use in dementia in the community will be carried out. It is anticipated that this will highlight (1) investigations on impact of AT use on carers, (2) outcome measures and experience questionnaires that have been used and (3) the types of studies carried out so far on this topic. The results from the review will be presented in a summary matrix of common types (e.g. mobile phones, alarms) and uses (e.g. communication, safety, personal care) of AT in dementia care and also identify AT that is not usually available through government or health system funding.

**Systematic review registration:**

PROSPERO CRD42017082268.

**Electronic supplementary material:**

The online version of this article (10.1186/s13643-019-1081-x) contains supplementary material, which is available to authorized users.

## Background

Dementia is becoming increasingly prevalent as the population ages and has been declared a public health priority [1]. There are an estimated 46.8 million people living with dementia globally, and the number is likely to double by 2035 [2]. Most of the care for persons with dementia living at home is provided by informal cares [3]. In the UK, there are over 700,000 informal carers for persons living with dementia [4]. Reports estimate the value of time given by informal carers to persons with dementia at approximately £12.4 billion a year for the UK [4, 5], and the global economic cost of dementia is estimated to be over US$ 604 billion [6]. In England, about 15% of dementia carers say they are not in work because of their caring responsibilities and around 39% of carers spend more than 100 h per week caring for a person with dementia [7].

Caring for an adult with dementia impacts on psychological, financial, economic and physical wellbeing of the carer [8–12]. Without support from informal carers, any formal support system for a person with dementia is likely to collapse. Caring for a person with dementia is not a static process [13], there is currently no cure for dementia and it is progressive; the trajectory of progress is uncertain, and carers are faced with the complexity of not knowing how quickly the dementia will progress. Therefore, it is vital to support carers in their experiences of providing care. In the UK, a national strategy for carers [14–16] is committed to supporting carers at an early stage and recognising the value of their contribution. Furthermore, the Care Act [17] sets out requirements for assessment and support centred around carers’ wellbeing. These, however, are poorly adopted. Stretched health and care resources necessitate alternative and innovative ways to providing care for persons with dementia [18]. Assistive technology has been suggested as a means to support persons with dementia and their carers to stay independent in the community [19, 20]. Assistive technology (AT) can be defined as ‘any item, piece of equipment, product or system that is used to increase, maintain or improve the functional capabilities and independence of people with cognitive, physical or communication difficulties’ [21].

AT can be specialist equipment that is prescribed by a healthcare professional or available ‘off-the shelf’. AT can be products such as clocks, medication dispensers, smartphone apps, robotic vacuum cleaners, smart gas meters, social assistive robots, communication books, navigation systems, falls and motion detectors and door exit alarms [22–24]. Use of AT by persons with dementia may by extension also benefit the carer [18, 25], which could potentially increase support from carers and alleviate some of the burden of caregiving [23, 26–28]. AT potentially assists carers to address the increased level of responsibility whilst caring for a person with dementia [29]. Future generations will be more familiar with technology with people having lived their entire lives influenced by it. With advances in the Internet of Things and artificial intelligence, AT is likely to become more personalised to individual needs and user requirements [30]. Home is an important and special place when ageing. Living at home brings with it a sense of security and freedom [31, 32], and persons with dementia will strive to live at home for as long as possible. With technological advances, AT would be one way of assuring this continues to happen. Carers of persons with dementia are in the unique position of using their perceptions regarding AT to suggest or even decide on the access and use of AT [33, 34].

## Rationale for this review

Currently, AT is being viewed as a panacea for reducing carer burden [32, 35]; multiple studies are investigating how AT can support persons with dementia [19, 36]. Carers could be using the AT together with the person with dementia and/or be looking after a person with dementia, who uses AT independently of the carer. However, little is known about the experiences of carers using AT and what impact AT has on carer outcomes [37]. This review aims to fill the gap in the literature that so far has predominantly looked at AT from the perspective of persons with dementia and its use within institutional settings [18, 38–40]. We seek to answer research questions regarding experiences of informal carers using various AT and their effectiveness. This information would benefit carers and persons with dementia and healthcare professionals who prescribe and set up AT solutions at home as well as industry and AT developers. The results from the review will result in a summary matrix of common types (e.g. mobile phones, alarms) and uses (e.g. communication, safety, personal care) of AT in dementia care. This matrix will be further refined in a consensus meeting with stakeholders, including carers and health professionals who usually prescribe AT.

## Review aim and questions

This review will aim to:

1. Identify the types and uses of AT in dementia
2. Describe the effectiveness of AT for burden, wellbeing and quality of life of informal carers of people with dementia living at home
3. Describe carers’ experiences of AT use in dementia
4. Determine the aspects of AT that are valued and work well for carers by integrating (2) and (3) as above.

The following information will also be reported: What was the profile of informal carers and persons with dementia in the studies? What was the range of outcome measures used to report on burden, quality of life and wellbeing in the included studies? How is the caregiver/care receiver relationship reported?

## Methods/design

This protocol has been prepared following the Preferred Reporting Items for Systematic Review and Meta-Analysis Protocols (PRISMA-P) guidelines for systematic review protocols [41]. (The PRISMA-P checklist is included as Additional file 1). The review protocol has been registered with the International Prospective Register of Systematic Reviews PROSPERO (CRD42017082268).

This review will gather evidence on the effectiveness and informal carers’ experience when using AT to support persons with dementia at home including perceived burden, quality of life and wellbeing with use of AT at home.

### Inclusion/exclusion criteria

#### Types of participants

Studies that include informal carers who provide care for a person with dementia at home without being reimbursed will be included. Care could be supporting a person with dementia physically, emotionally, financially or socially and be provided by a relative, a friend or a neighbour of the person with dementia. There will be no restrictions regarding sex, living arrangements or ethnic background. Studies reporting on informal carers who provide support to a person with dementia receiving care in hospital and/or long-term institutions, informal carers who are younger than 18 years of age and formal/paid carers will be excluded.

#### Types of studies

To answer the review questions, both quantitative and qualitative study designs will be included. Restriction will not be placed on the identification of studies by design or methods. The full-text article or report of the study needs to be available (i.e. abstracts will be excluded). We will not be including other reviews; however, we will check references within identified existing reviews on dementia, informal carers and AT to ensure that all relevant studies have been located. Letters to the editor, abstract and conference proceedings and book reviews will not be included. Study protocols and theses/dissertations will also be excluded.

#### Types of assistive technology

For this review, studies that evaluate AT use in dementia involving informal carers will be included. AT will be defined as any advanced electronic equipment, which can be used to enhance support and care, act as a prompt for intervention by carers, monitor welfare and assist in communication and leisure activities for a person with dementia. This AT can be stand alone or be part of an integrated system and can be stationary or mobile. The focus of most research studies invariably is on the person with dementia, but any study that reports on effects or experiences of AT use on informal carers will be included. Studies that report solely on AT use for persons with dementia without including informal carers will be excluded, as will studies that focus only on electronic therapeutic interventions that are not AT (e.g. computer-based education or support for carers).

#### Comparators

For appropriate study designs, the control interventions could be ‘care (or treatment) as usual’ or non-technological or other nonpharmacological interventions such as monitoring and support at home by paid carers and interventions that use non-electronic assistive devices such as dosette boxes.

#### Outcome measures

##### Primary

For evaluation of effectiveness, carer burden, quality of life, and wellbeing will be included. For evaluation of experience, all reported or observed experiences of usefulness, benefits and disadvantages of AT and impact on caregiver/care receiver relationship will be included.

##### Secondary

For evaluation of effectiveness, carer self-esteem and feeling of competence and reported environmental changes to the home as a result of AT use will be considered. For evaluating experience, barriers and facilitators, user-friendliness and support required to use AT will be considered.

A summary of the criteria for inclusion and exclusion of studies for this systematic review is given in Additional file 4: Table S1.

### Search strategy

The search strategy has been developed in collaboration with a Bodleian medical library librarian at the University of Oxford.

Searches will be carried out on:

A. Databases

The databases included were MEDLINE (Ovid) from 1946 to present, EMBASE from 1974 to present, PsycINFO from 1806 to present, AMED 1985 to present, CINAHL from 1981 to present, Database of Abstracts of Reviews of Effects (DARE), OT seeker and The Cochrane Library of Systematic Reviews. The search will include studies within ALOIS (from database inception to present). ALOIS is maintained by the Trials Search Coordinator of the Cochrane Dementia Group and contains dementia and cognitive improvement studies identified from various databases and trial registries.

B. Unpublished literature

The International Standard Randomised Controlled Trials Number (ISRCTN) registry [42] and the National Institutes of Health Clinical Trials Database [43] will be searched for information on unpublished trials, and OpenGrey will be searched for conference papers, official publications and other types of grey literature. In addition, organisations within national and international settings, including AGE-WELL NCE (Aging Gracefully across Environments using Technology to Support Wellness, Engagement and Long Life) in Canada and Rehabilitation Engineering and Assistive Technology Society for North America (RESNA) will be contacted. Searches within these databases will be used to identify additional studies and authors to contact for full-text reports.

C. Manual searches of reference lists will also be conducted to identify relevant research studies.

Searches will not be limited by quantitative or qualitative filters, but due to funding constraints, only English language or those translated to English language will be included. We will however scan references of potential papers in languages other than English and list these in the review to provide a more complete picture of potential evidence, to inform future updates of the review. No date limits will be applied. The search strategy for the search on MEDLINE (Ovid) database is provided (Additional file 2).

### Screening

Electronic search results will be downloaded into Covidence software [44]. Covidence is a web-based software platform that streamlines the production of systematic reviews. Duplicates will be removed using the software. Authors VS and MP will independently screen all titles and abstracts for eligibility against our inclusion/exclusion criteria. Any discrepancies will be resolved by mutual discussion. For studies that have insufficient information from the title and abstract, full text will be retrieved to determine inclusion. Studies marked for possible inclusion will undergo a full-text review. At full-text review, if both VS and MP agree that a study does not meet the full eligibility criteria, the study will be excluded. Any disagreement will be resolved by discussion and in consensus with the third author CJ. Reasons for exclusion of the full-text studies will be documented and listed separately.

### Data extraction

A bespoke data extraction form (Additional file 3) developed by the authors will be used and initially piloted on a sample of studies to refine the form. Data will be extracted differently for the effectiveness and the experience evaluations from quantitative and qualitative studies respectively, and appropriate columns will be filled for mixed method studies.

### Evaluation of effectiveness

Data extraction from quantitative studies will be based on the recommended items from the Cochrane handbook for systematic reviews of interventions [45]. Information on citation including authors and contact details, study design, duration, number of participants, setting, participant gender, age and ethnicity, country where the study took place, relationship status to the person with dementia, interventions, outcome measures or scaled used, time points of data collection, missing participants and key conclusions from the study authors will be extracted.

### Evaluation of experience

In addition to collecting information from qualitative studies on citation, author contact details, study design, duration, setting, participant information, country and time points when information was collected, VS will extract data based on participants’ quotes and study authors’ commentaries. MP will check all extracted data for accuracy and completeness. Any disagreement will be resolved by discussion with CJ.

### Risk of bias assessment

The Mixed Methods Appraisal Tool [46–48] for assessing bias will be used for risk of bias assessment. This tool is designed for the appraisal stage of systematic mixed studies reviews, i.e. reviews that include qualitative, quantitative and mixed methods studies. The selected studies will be critically evaluated by VS and discussed with MP and CJ with any discrepancies resolved through discussion. Authors’ conclusions on the quality of the included studies will be part of the summary of included studies.

### Data synthesis

Where possible, quantitative results of studies will be pooled based on outcome measures and we will show forest plots and estimate pooled effects. Qualitative results will be synthesised using thematic synthesis [49]. Our preliminary searches and other reviews in AT have shown studies are heterogeneous; in this case, a descriptive summary and narrative synthesis [50] of the included studies will be provided. Where data is available, a subgroup analysis using chi-squared test for sub-group differences to assess for subgroup interactions based on device types, uses, living arrangements, adverse events and demographics of carers (country, age, gender, relationship, ethnicity etc.) will be done. VS will extract data and summarise the results. MP will review and highlight any discrepancies, and outstanding issues will be resolved through mutual discussion and involvement of CJ as necessary.

## Potential limitations

We anticipate certain limitations to our review. Due to the prospect that there will be a widespread range of AT, reported outcomes and outcome measures used within the studies, there will be widespread heterogeneity in the included studies. Due to financial constraints, we are not including languages other than English within this review and this could potentially miss some suitable studies. We are also not including unpublished data within this systematic review due to the potential of low quality of data from non-peer-reviewed sources.

## Discussion

The main aim of this review is to describe informal carers’ perception and experience of AT use in dementia care. The results of the review are expected to inform a summary matrix of common types (e.g. mobile phones, alarms) and uses (e.g. communication, safety, personal care) of AT in dementia care. The review will also identify commonly used outcome measures that assess informal carer burden and quality of life in conjunction with AT use in dementia. The inclusion of quantitative and qualitative study designs will help understand the breadth and depth of informal carers’ experience of AT use for a person with dementia. The review will highlight investigations on the impact of AT use on informal carers and commonly used outcome measures and experience questionnaires that have been used within this research area. The findings from this review will be published and disseminated (journals, conferences and social media), and it is anticipated that the findings will be useful for healthcare professionals, commissioners and AT developers to inform better provision of care in the community for carers of persons with dementia.

## Additional files

Additional file 1: PRISMA-P checklist. (DOCX 35 kb)

Additional file 2: MEDLINE (OVID) search strategy for this review. (DOCX 13 kb)

Additional file 3: Data extraction form for this review. (DOCX 15 kb)

Additional file 4: Table of inclusion and exclusion criteria for this review. (DOCX 15 kb)

## Data Availability

Not applicable.

## Abbreviations

ALOIS: ALOIS, named after Alois Alzheimer, is a register of dementia studies maintained by the Cochrane Dementia and Cognitive Improvement Group
AMED: Allied and Complementary Medicine Database
AT: Assistive technology
CINAHL: Cumulative Index of Nursing and Allied Health Literature
PRISMA-P: Preferred Reporting Items for Systematic Reviews and Meta-Analyses Protocols
PROSPERO: International Prospective Register of Systematic Review
