# Supplementary material for: Informal carers’ experience and outcomes of assistive technology use in dementia care in the community: a systematic review protocol
Source: Syst Rev. 2019 Jul 3;8:158. doi: 10.1186/s13643-019-1081-x (PMC6610817; doi:10.1186/s13643-019-1081-x)
Supplement: Supplementary file 2 — MEDLINE (OVID) search strategy for this review. (DOCX 13 kb) [file 13643_2019_1081_MOESM2_ESM.docx]

**Informal carers’ experience and outcomes of assistive technology use in dementia care in the community: A systematic review protocol.**

Database: Ovid MEDLINE(R) Epub Ahead of Print, In-Process & Other Non-Indexed Citations, Ovid MEDLINE(R) Daily and Ovid MEDLINE(R) <1946 to Present>

Search Strategy:

| # ▲ | Searches |  |
| --- | --- | --- |
| 1 | exp Dementia/ or dement*.mp. |  |
| 2 | (lewy bodies or alzheimer*).mp. |  |
| 3 | exp Wernicke Encephalopathy/ or wernicke encephalopath*.mp. |  |
| 4 | exp Korsakoff Syndrome/ or korsakoff.mp. |  |
| 5 | 1 or 2 or 3 or 4 |  |
| 6 | Technology/ or technolo*.mp. |  |
| 7 | (informat* adj2 communicat*).mp. |  |
| 8 | ict.mp. |  |
| 9 | Information Systems/ or Telemedicine/ or Monitoring, Ambulatory/ |  |
| 10 | (telemedicine or tele medicine or telehealth or tele health or telemonitor* or tele monitor* or ehealth or e-health or mhealth or m-health).mp. |  |
| 11 | Telephone/ or remote consultation/ |  |
| 12 | "Activities of Daily Living"/ or Self-Help Devices/ or assistive.mp. |  |
| 13 | device*.mp. |  |
| 14 | self care.mp. or Self Care/ |  |
| 15 | self help.mp. |  |
| 16 | (gero* adj2 tech*).mp. |  |
| 17 | robo*.mp. or Robotics/ |  |
| 18 | (assist* adj2 (aid? or device?)).mp. |  |
| 19 | "Equipment and Supplies"/ or Equipment Design/ or equipm*.mp. |  |
| 20 | Signal Processing, Computer-Assisted/ or Clothing/ or wearable*.mp. |  |
| 21 | 6 or 7 or 8 or 9 or 10 or 11 or 12 or 13 or 14 or 15 or 16 or 17 or 18 or 19 or 20 |  |
| 22 | Caregivers/ or caregiv*.mp. |  |
| 23 | (relatives or family or families).mp. or exp Family/ |  |
| 24 | friend*.mp. or Friends/ |  |
| 25 | (neighbour? or neighbor?).mp. |  |
| 26 | Spouses/ or spous*.mp. |  |
| 27 | (wife or wives).mp. |  |
| 28 | husband?.mp. |  |
| 29 | (couple? or partner?).mp. |  |
| 30 | son?.mp. |  |
| 31 | daughter?.mp. |  |
| 32 | Home Nursing/ or carer*.mp. |  |
| 33 | Home Care Services/ or home.mp. |  |
| 34 | 22 or 23 or 24 or 25 or 26 or 27 or 28 or 29 or 30 or 31 or 32 or 33 |  |
| 35 | 5 and 21 and 34 |  |
