# Supplementary material for: Informal carers’ experience and outcomes of assistive technology use in dementia care in the community: a systematic review protocol
Source: Syst Rev. 2019 Jul 3;8:158. doi: 10.1186/s13643-019-1081-x (PMC6610817; doi:10.1186/s13643-019-1081-x)
Supplement: Supplementary file 3 — Data extraction form for this review. (DOCX 15 kb) [file 13643_2019_1081_MOESM3_ESM.docx]

# Informal carers’ experience and outcomes of assistive technology use in dementia care in the community: A systematic review protocol.

# Data extraction form: Quantitative studies

| Data to be extracted | Study Data |
| --- | --- |
| Publication Characteristics |  |
| Title |  |
| Publication type [Peer reviewed journal article, Trial, SR] |  |
| Year of Publication |  |
| Geographical location of study |  |
| Author (s) |  |
| Author contact details |  |
| Study characteristics |  |
| RCT, Case Control, Feasibility etc. |  |
| Study duration (longitudinal, survey, pilot etc.) |  |
| Participant characteristics (for informal carers) |  |
| Age [in years] |  |
| Gender [M/F] |  |
| Relationship with person with dementia |  |
| Ethnicity |  |
| Type/severity of dementia (of person being cared for) |  |
| Co-morbidities recorded |  |
| Living arrangements (with patient with dementia or away) |  |
| Other sub-groups reported |  |
| Intervention characteristics |  |
| Duration of participation [hours or days] |  |
| Number of participants in each group |  |
| AT description |  |
| Suggested use of AT |  |
| Number of hours AT used [hours] |  |
| Mobile/Fixed device |  |
| Stand-alone/system based AT |  |
| Off- the shelf/bespoke AT |  |
| Cost reported [Yes/No] |  |
| Who paid for AT? [Self, Trial, Healthcare provider] |  |
| Adherence to protocol [Yes, No] |  |
| Comparator [If listed] |  |
| Loss to follow-up/withdrawals |  |
| Outcome characteristics |  |
| Primary outcomes reported |  |
| Secondary outcomes reported |  |
| Measures used for Burden, QoL, wellbeing |  |
| Are the measures used validated/peer-reviewed? |  |
| Other Measures used |  |
| Are the measures validated/peer-reviewed? |  |
| Missing data reported |  |
| How was missing data handled? |  |
| Time points of data collection |  |
| Other characteristics |  |
| Conflicts of interests |  |

# Data extraction form: Qualitative studies

| Data to be extracted | Study Data |
| --- | --- |
| Publication Characteristics |  |
| Title |  |
| Publication type |  |
| Year of Publication |  |
| Geographical location of study |  |
| Author (s) |  |
| Author contact details |  |
| Study characteristics |  |
| Study Aim |  |
| Theoretical perspective of the research |  |
| Data collection approach - Interview, Focus group, Observation etc.? |  |
| Study duration (longitudinal, pilot etc.) |  |
| Participant characteristics (for informal carers) |  |
| Appropriate sample included? |  |
| Age [in years] |  |
| Gender [M/F] |  |
| Relationship with person with dementia |  |
| Ethnicity |  |
| Type/severity of dementia (of person being cared for) |  |
| Co-morbidities recorded |  |
| Living arrangements (with patient with dementia or away) |  |
| Other sub-groups reported |  |
| Intervention characteristics |  |
| Duration of participation [hours or days] |  |
| Number of participants |  |
| AT description |  |
| Suggested use of AT |  |
| Number of hours AT used [hours] |  |
| Mobile/Fixed device |  |
| Stand-alone/system based AT |  |
| Off- the shelf/bespoke AT |  |
| Cost reported [Yes/No] |  |
| Who paid for AT? [Self, Trial, Healthcare provider] |  |
| Loss to follow-up/withdrawals |  |
| Outcome characteristics |  |
| Data analysis (clear, systematic, rigorous etc.) |  |
| Key themes reported:First Order (Participant quotes):Second order (Author statements):Third order (summary): |  |
| Time points of data collection |  |
| Relevance of findings |  |
| Author’s Key conclusions |  |
| Generalisability |  |
| Other characteristics |  |
| Conflicts of interests |  |
