# Supplementary material for: Informal carers’ experience and outcomes of assistive technology use in dementia care in the community: a systematic review protocol
Source: Syst Rev. 2019 Jul 3;8:158. doi: 10.1186/s13643-019-1081-x (PMC6610817; doi:10.1186/s13643-019-1081-x)
Supplement: Supplementary file 4 — Table of inclusion and exclusion criteria for this review. (DOCX 15 kb) [file 13643_2019_1081_MOESM4_ESM.docx]

Informal carers’ experience and outcomes of assistive technology use in dementia care in the community: A systematic review protocol.

Table 1: Inclusion and Exclusion criteria for this review

| Category | Inclusion | Exclusion |
| --- | --- | --- |
| Types of Participants | Informal carer of a person with dementia - Relative, Friend, Neighbour | - Formal/Paid carers - Informal carers aged less than 18 years |
| Types of studies | - Quantitative and Qualitative studies - Studies in which electronic Assistive Technology is used by or together with a person with dementia | - Studies without full text reports despite contacting authors - Letters to the editor - Any type of reviews - Book reviews, books or book chapters - Abstracts and conference proceedings - Study protocols - Thesis/Dissertations |
| Assistive Technology | Advanced electronic equipment, which can be used for, or together with, a person with dementia to:   - Enhance support and care, - Act as a prompt for intervention by carers, - Monitor welfare - Assist in communication - Assist in Leisure activities | - Non electronic AT. - Computer, smartphone or telephone delivered interventions such as education for carers or psychological support interventions. - Prototypes of Assistive Technology - Opinions on Assistive Technology use rather than actual use. |
| Carer experience and outcomes | Studies where either experiences or outcomes of carers are reported.  Carer outcomes include   - burden - quality of life - wellbeing - Self-esteem - Anxiety / depression - Distress - Feelings of competence - Impact on carer and person with dementia relationship   Experiences with AT include:   - Usefulness - Benefits and disadvantages and - Barriers and facilitators - User-friendliness - Support/Assistance required in using AT - Reported environmental changes to the home as a result of AT use. | - Reports of experience or outcomes of the person with dementia only - Reports of experience or outcomes where informal carers act as a proxy for the person with dementia - Studies collecting data on carer views of electronic AT under development - Studies collecting data on carers general views or attitudes on AT but were no AT was used. |
| Setting | - Person with dementia living in their own home in the community - Carer is either living with or away from the person with dementia they care for | - Person with dementia in a hospital setting - Person with dementia in a long-term institution (i.e. Nursing homes/residential homes/Sheltered housing) |
| Language | English |  |
| Geography | All |  |
